# Disclosure of Amyloid Status for Risk of Alzheimer Disease to Cognitively Normal Research Participants With Subjective Cognitive Decline: A Longitudinal Study

**DOI:** 10.1177/1533317520904551

**Published:** 2020-02-13

**Authors:** Taisei Wake, Hajime Tabuchi, Kei Funaki, Daisuke Ito, Bun Yamagata, Takahito Yoshizaki, Tadaki Nakahara, Masahiro Jinzaki, Haruo Yoshimasu, Iori Tanahashi, Hiroumi Shimazaki, Masaru Mimura

**Affiliations:** 1 Department of Psychiatry, Saitama Medical Center, Saitama Medical University, Saitama, Japan; 2 Department of Neuropsychiatry, Keio University School of Medicine, Tokyo, Japan; 3 Department of Neurology, Keio University School of Medicine, Tokyo, Japan; 4 Department of Diagnostic Radiology, Keio University School of Medicine, Tokyo, Japan

**Keywords:** subjective cognitive decline, disclosure, amyloid imaging, ethics, Alzheimer disease

## Abstract

This study aimed to investigate the long-term impacts of disclosing amyloid status for a risk of Alzheimer disease (AD) to cognitively normal research participants with subjective cognitive decline (SCD), which represents an initial manifestation of AD. Forty-two participants were classified as the amyloid-positive (*n* = 10) or amyloid-negative (*n* = 32) groups. We assessed symptoms of anxiety, depression, and test-related distress at 6, 24, and 52 weeks after results disclosure. No difference was found over time in anxiety, depression, and test-related distress in either group. Although no significant differences were observed between groups in anxiety or depression, the amyloid-negative group had a significantly higher level of test-related distress than the amyloid-positive group at 52 weeks. Disclosing amyloid status to cognitively healthy research participants with SCD did not cause significant long-term psychological risks. However, a theoretical spectrum of subjective concern may exist about cognitive decline in amyloid-negative individuals.

## Introduction

Despite widespread use of amyloid imaging to predict future risk of Alzheimer disease (AD), understanding the psychological impact of disclosing imaging results to cognitively normal research participants has not been studied in detail.^1^ In particular, the safety of sharing results among healthy participants with subjective cognitive decline (SCD) has rarely been investigated.

Subjective cognitive decline refers to persistent self-perceived cognitive decline in the absence of objective neuropsychological impairments.^2,3^ Neuroimaging research suggested associations between SCD- and AD-related brain alternations in otherwise cognitively healthy adults, including changes in the cholinergic-basal forebrain nucleus,^4^ medial temporal atrophy,^5,6^ glucose metabolism reduction,^7^ and amyloid-beta (Aβ) aggregation as evaluated with positron emission tomography (PET).^8,9^ As such, SCD is receiving increasing attention as the first symptomatic manifestation of AD and other dementias.^10-12^

The impact of informing healthy research participants with SCD about amyloid PET results for the risk for AD warrants further attention. A recent study showed that anxiety symptoms were associated with greater cognitive decline in the amyloid-positive group in the preclinical stage, even without knowing their amyloid status.^13^ Given that associations between SCD and higher levels of anxiety have been demonstrated,^14-16^ and given that there is a lack of scientific knowledge on how to appropriately interpret and effectively communicate the uncertainty of the PET results,^17^ disclosure of amyloid-positive findings to those with SCD may increase anxiety and consequently result in Aβ-related cognitive decline.

Nevertheless, amyloid imaging has been used as a screening tool in antiamyloid trials to identify amyloid-positive participants to be enrolled in the study, and disclosure of results to cognitively normal participants is incorporated into the study design.^18^ A few studies indicated the safety of disclosure on cognitively normal participants,^19,20^ though they did not make any differentiation for those with SCD, which may represent a possible high-risk group for emotional harm. Improved understanding is needed to investigate the impact of disclosing PET results to these participants.

Our preliminary study revealed that no significant short-term psychological risks were found by disclosing amyloid status to cognitively normal persons with SCD.^21^ However, that study was limited by its short-term status. In the current study, we examined the safety of disclosure over the long-term to extend the results of our preliminary study. We measured the level of anxiety, depression, and test-related distress at baseline, 6, 24, and 52 weeks after disclosure.

## Method

### Recruitment

As previously described,^21^ we conducted clinical observation and self-report-based quantitative research as part of our institutionally approved study aimed at predicting abnormal accumulation of Aβ by neuropsychological assessments and regional cerebral blood flow in cognitively normal adults with SCD.^22^ Participants were recruited into the current study from the memory clinic at Keio University Hospital located in central Tokyo from May 2015 to September 2016.

### Definition of SCD

Research criteria for SCD in preclinical AD were based on consensus terminology and a conceptual framework of an international working group named Subjective Cognitive Decline Initiative (SCD-I)^2,23^ and were defined as (*a*) persistent self-perceived cognitive decline and (*b*) normal performance on standardized objective neuropsychological tests adjusted by age, gender, and education. Another equally important core feature of SCD was also required for eligibility based on the more refined criteria called SCD-plus: (*c*) concerns (worries) associated with SCD that may lead to seeking medical help.^15,24,25^

Potential participants visited the university hospital with concerns regarding self-perceived persistent cognitive decline. As previous studies suggested, worry about memory is the better predictor of underlying AD than other cognitive domains.^23,25^ Subjective cognitive decline was assessed by directly asking “Do you feel like your memory is worsening?” (3 possible answers were no; yes, but SCD does not worry me; and yes, this worries me) (SCD criteria a and c).^2^ All participants underwent a diagnostic evaluation performed on a routine basis at the memory clinic including neuropsychological test batteries, magnetic resonance imaging (MRI), and single photon emission computed tomography (SPECT) of the brain. The neuropsychological batteries consist of the following 8 tests: Mini-Mental State Examination (MMSE),^26^ Raven Colored Progressive Matrices,^27^ Rey Auditory Verbal Learning Test,^28^ Rey-Osterrieth Complex Figure Test,^29,30^ Logical Memory subtest of the Wechsler Memory Scale–Revised (WMS-R),^31^ Trail Making Test (parts A and B),^32^ and Word fluency (initial letter and category).^33^ Subjective cognitive decline in preclinical AD occurs, by definition, in absence of detectable abnormalities of these conventional tests (SCD criteria b).

### Other Inclusion and Exclusion Criteria

Participants of the parent and current studies were required to meet other inclusion criteria as follows: aged 65–85 years, MMSE score of ≥27, Logical Memory subtest (immediate and delayed recall) from the WMS-R score within normal range using education-adjusted cutoff scores, Clinical Dementia Rating^34^ = 0, and Hachinski Ischemic Score ≤ 4. Exclusion criteria were as follows: diagnosed with neurologic or neurodegenerative disorders other than preclinical AD; having any medical condition known to affect cognitive function; had a history of schizophrenia, bipolar disorder, depression, or anxiety disorder within the past 1 year; or a history of chronic alcohol or drug abuse/dependence within the past 2 years. Additional scales assessing geriatric depression and apathy were performed at enrollment.

### Psychoeducation Before Consent

At the time of informed consent for the current study, all participants underwent a psychoeducational session performed by the psychiatrists on the research team. Supplemental material was provided in the session to describe (*a*) the clinical meaning of the preclinical stage in the course of AD, (*b*) the findings which suggested that abnormal accumulation of aggregated Aβ in the brain is believed to increase risk of developing AD, (*c*) the uncertainty and limitations of the new technology, and (*d*) the potential benefits and harms in receiving the results of the PET scan based on previous studies. Before giving consent, all participants verbally showed an adequate level of understanding about the implication of Aβ positivity and negativity. Participants were asked on the consent form whether they preferred PET scan results to be disclosed or not. Only those who agreed to receive results were included in this study.

### Amyloid Imaging Procedure

Amyloid status was evaluated using amyloid PET [^18^F] AV1 (florbetaben). Positron emission tomography images were interpreted by 2 nuclear medicine experts certified by the manufacturer (Primal Imaging GmbH, Berlin, Germany). Amyloid-β positivity and negativity were discriminated by assessing tracer uptake in the gray matter in accordance with the guidelines.^35^ Positron emission tomography results were disclosed to each participant by 2 experienced psychiatrists on the study team.

### Outcome Measures

Baseline anxiety and depression levels of all the participants were obtained at enrollment (predisclosure) as measured by the State-Trait Anxiety Inventory (STAI)^36^ and the Beck Depression Inventory-II (BDI-II).^37^ The 2 measurements and the Impact of Event Scale–Revised (IES-R)^38^ were performed at 6, 24, and 52 weeks after disclosure. The IES-R is a 22-item self-reported measure with scores ranging from 0 to 88; higher scores indicate stronger test-related distress. We prespecified cutoff scores in measurements of state anxiety, depression, and test-related distress as 54/55, 17/18, and 24/25, respectively, based on previous findings about optimal cutoff values to detect clinically significant symptoms among geriatric populations.^38-40^ All measurement scales were mailed to participants and returned at each follow-up time point.

The primary outcomes of the current study were changes over time in participants’ anxiety, depression, and test-related distress levels, as measured by the STAI (state), BDI-II, and IES-R, respectively. The secondary outcome was to compare the amyloid-positive group with the amyloid-negative group in terms of scores on each of the 3 measurements at each follow-up time.

### Statistical Analysis

Independent sample *t* tests were used to assess group differences in demographic and descriptive variables. To describe a central tendency and dispersion of the scores of the STAI (state), BDI-II, and IES-R, percentiles were calculated for each measurement. Given the relatively small sample size, we performed a Wilcoxon signed-rank test to examine within-group differences and Welch’s analysis of variance (ANOVA) for between-group differences on the 3 measures. All analyses were 2-tailed, and *P* < .05 was considered to indicate statistical significance. Statistical tests were performed using IBM SPSS Statistics for Mac, version 25.0.

### Safety Monitoring

Participants with amyloid-positive PET results were strongly recommended to arrange long-term follow-up visits at the memory clinic to evaluate their cognitive function and the psychological effect of the disclosure of amyloid status. Participants with amyloid-negative results were also monitored regarding changes in anxiety, depression, and test-related distress at each follow-up point, as well as during additional follow-up visits, if requested by participants. The study was approved by the ethics committee of Keio University School of Medicine (IRB number: 20130509). All participants provided written informed consent.

## Results

### Demographics

A total of 43 people met the inclusion criteria and agreed to participate in the study. All participants provided consent to undergo amyloid PET imaging and have their results disclosed to them. One participant voluntarily withdrew from the study before the PET scan because of personal health issues. The remaining 42 participants had amyloid imaging and completed the follow-up evaluations through 52 weeks (Table 1). Of these, 10 participants (24%) had amyloid-positive results. No significant differences were observed between the amyloid-positive group and amyloid-negative group with regard to age, education, or scores on the MMSE, Logical Memory, STAI (state and trait), and BDI-II. No participant reported a history of schizophrenia, bipolar disorder, depression or anxiety disorder, and chronic alcohol or drug abuse/dependence.

**Table 1.** Demographic Data at Baseline (Predisclosure).^a^

	Amyloid-Positive Group (*n* = 10)	Amyloid-Negative Group (*n* = 32)	Statistics^b^		
			*t*	df	*P* Value
Age (years)	75.5 ± 4.7	74.1 ± 4.8	0.80	40	.43
Female, *n* (%)	6 (60.0)	16 (50.0)			
Mean education (years)	15.0 ± 1.7	15.0 ± 2.2	−0.12	40	.90
MMSE	29.2 ± 1.1	29.2 ± 1.0	−0.05	40	.96
LM (immediate recall)	13.3 ± 2.4	12.8 ± 3.4	−0.50	40	.62
LM (delayed recall)	10.5 ± 2.2	11.7 ± 3.0	0.52	40	.61
RCPM	30.4 ± 2.8	31.7 ± 2.2	1.48	40	.15
RAVLT (first to fifth sum)	53.7 ± 7.6	51.2 ± 9.8	−0.37	40	.47
ROCFT (copy)	35.2 ± 1.3	35.6 ± 0.9	1.07	40	.29
ROCFT (delay)	21.0 ± 5.4	20.6 ± 5.6	−0.18	40	.86
TMT B − TMT A/TMT A	0.39 ± 0.37	0.24 ± 0.38	−1.08	40	.29
Verbal fluency (initial letter)	29.6 ± 7.8	26.8 ± 7.7	−1.01	40	.32
Verbal fluency (category)	42.4 ± 7.6	41.8 ± 9.6	−0.17	40	.87
GDS	3.8 ± 3.2	4.3 ± 3.6	−0.43	40	.67
Apathy scale	8.8 ± 6.1	10.6 ± 5.6	−0.85	40	.40

Abbreviations: GDS, Geriatric Depression Scale; LM, Logical Memory subtest of the Wechsler Memory Scale; MMSE, Mini-Mental State Examination; RAVLT, Rey Auditory Verbal Learning Test; RCPM, Raven Colored Progressive Matrices; ROCFT, Rey-Osterrieth Complex Figure Test; TMT, Trail Making Test.

^a^ Values are *n* (%) or mean ± SD.

^b^ Independent *t*- test.

### Individual Participant-Level Analysis

Table 2 shows percentiles of the STAI (state), BDI-II, and IES-R scores in the amyloid-negative group. Medians of the 3 measurements at all time points were well below the prespecified cutoff points of 54/55, 17/18, and 24/25, respectively. As individual participant-level analysis, paired changes were calculated by subtracting scores at predisclosure (6-week follow-up for IES-R) from scores at 52-week follow-up in each individual participant. It was suggestive, but not statistically significant as described below, that individual paired change scores in the amyloid-negative group at the 75th percentile in the STAI (state) and IES-R were higher than the ones in the amyloid-positive group.

**Table 2.** Percentiles of the STAI, BDI-II, and IES-R Scores.^a^

	Amyloid-Positive Group (*n* = 10)			Amyloid-Negative Group (*n* = 32)		
	25%	50%	75%	25%	50%	75%
STAI score (state)						
Predisclosure	31.0	36.5	44.0	30.0	41.0	47.0
52 weeks	31.0	42.0	49.0	34.0	44.0	54.0
Paired change	−4.0	+0.5	+3.0	−2.0	+2.0	+11.5
BDI score						
Predisclosure	8.0	9.0	13.0	3.5	11.5	17.5
52 weeks	5.0	7.0	11.0	6.5	12.5	19.0
Paired change	−5.0	−1.0	+5.0	−2.0	+1.0	+4.0
IES-R score						
6 weeks	1.0	6.5	11.0	2.5	10.5	21.5
52 weeks	3.0	5.5	14.0	1.0	15.0	30.5
Paired change	−4.0	+0.0	+3.0	−2.5	+0.5	+11.5

Abbreviations: BDI, Beck Depression Inventory-II; IES-R, Impact of Events Scale–Revised; STAI, State-Trait Anxiety Inventory.

^a^ Values are actual psychometric scale scores of each participant. Paired changes were calculated by subtracting scores at predisclosure (6-week follow-up for IES-R) from scores at 52-week follow-up in each individual participant.

### Within–Between-Group Differences: Anxiety Depression

**Figure 1.**
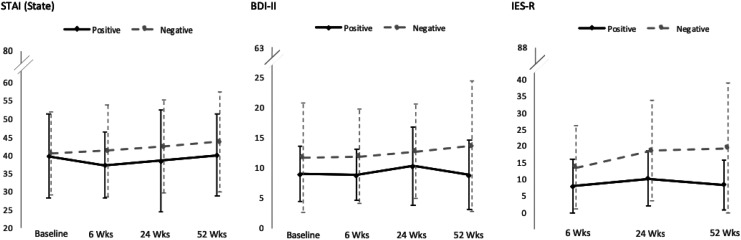
Mean scores of the STAI (state anxiety), BDI-II, and IES-R at each time point. BDI-II indicates Beck Depression Inventory-II; IES-R, Impact of Events Scale–Revised; Negative, amyloid-negative group (*n* = 32); Positive, amyloid-positive group (*n* = 10); STAI, State-Trait Anxiety Inventory.

Means of the STAI (state), BDI-II, and IES-R at all time points were well below the prespecified cutoff points of 54/55, 17/18, and 24/25, respectively (Table 3, Figure 1). Values of the Shapiro-Wilk test were below 0.05 in the STAI (state) score in the amyloid-positive group at 24 weeks after disclosure (*W*_10_ = 0.80, *P* = .02), in BDI-II scores in the amyloid-positive group at 24 weeks of follow-up (*W*_10_ = 0.82, *P* = .03), and in the amyloid-negative group at 52 weeks of follow-up (*W*_32_ = 0.84, *P* = .04). Also, the value of a Levene’s test was below 0.05 in the BDI score at baseline (*F*_1_ = 5.81, *P* = .02).

**Table 3.** Results of Follow-Up Assessments at 6, 24, and 52 Weeks After Disclosure (Postdisclosure).^a^

	Amyloid-Positive Group (*n* = 10)	Amyloid-Negative Group (*n* = 32)	Statistics^b^		
			*F*	df	Adjusted *P*
STAI score (trait)					
Predisclosure	40.9 ± 9.9	40.3 ± 11.7	0.04	1	1.00
STAI score (state)					
Predisclosure	39.8 ± 11.6	40.6 ± 11.5	0.02	1	1.00
6 weeks	37.3 ± 9.1	41.3 ± 12.7	1.19	1	1.00
24 weeks	38.5 ± 14.2	42.4 ± 12.9	0.60	1	1.00
52 weeks	40.1 ± 11.3	43.8 ± 13.8	0.72	1	1.00
BDI score					
Predisclosure	9.1 ± 4.5	11.8 ± 9.1	1.60	1	.86
6 weeks	8.9 ± 4.2	11.9 ± 7.8	2.50	1	.50
24 weeks	10.4 ± 6.5	12.8 ± 7.8	0.99	1	1.00
52 weeks	8.9 ± 5.7	13.7 ± 10.8	3.37	1	.30
IES-R score					
6 weeks	7.9 ± 8.1	13.6 ± 12.5	2.81	1	.32
24 weeks	10.1 ± 8.3	18.7 ± 15.2	5.22	1	.09
52 weeks	8.3 ± 7.5	19.3 ± 19.7	6.85	1	**.04**

Abbreviations: BDI, Beck Depression Inventory-II; IES-R, Impact of Events Scale–Revised; STAI, State-Trait Anxiety Inventory.

^a^ Values are *n* (%) or mean ± SD.

^b^ Welch’s ANOVA with robust tests of equality of means; significant values are highlighted in boldface.

Given that the assumption of normality was violated in multiple scores, the Wilcoxon signed-ranks test was performed. It indicated no significant differences between baseline and 52 weeks follow-up scores in the STAI (state) in the amyloid-positive group (*Z* = −0.12, *P* = .91) and amyloid-negative group (*Z* = −1.57, *P* = .12), or in the BDI in the amyloid-positive group (*Z* = −0.21, *P* = .84) and amyloid-negative group (*Z* = −1.30, *P* = .19). Similarly, robust tests of equality of means in the Welch ANOVA indicated no significant differences between groups at any time points in STAI (state) or BDI-II scores (Table 3).

### Within–Between-Group Differences: Test-Related Distress

Values of the Shapiro-Wilk test were below 0.05 in the STAI (state) score in the amyloid-positive group at 6 weeks after disclosure (*W*_10_ = 0.84, *P* = .04), and in the BDI-II scores at all time points in the amyloid-negative group (*W*_32_ = 0.90, *P* = .08; *W*_32_ = 0.93, *P* = .33; *W*_32_ = 0.87, *P* = .01, respectively). Also, the value of a Levene’s test was below 0.05 in the BDI scores at 24- and 52-week follow-up (*F*_1_ = 5.04, *P* = .03; *F*_1_ = 7.42, *P* = .01, respectively).

Given that the assumption of normality was violated in multiple scores, the Wilcoxon signed-ranks test was performed. It indicated no significant differences between 6- and 52-week follow-up scores in the IES-R scores in the amyloid-positive group (*Z* = −0.07, *P* = .94) and amyloid-negative group (*Z* = −1.52, *P* = .13). However, robust tests of equality of means in the Welch ANOVA indicated significant difference between groups at 52 weeks follow-up as shown in Table 3.

### Correlations Between Baseline Anxiety Level and Test-Related Distress Level at 52 Weeks

**Figure 2.**
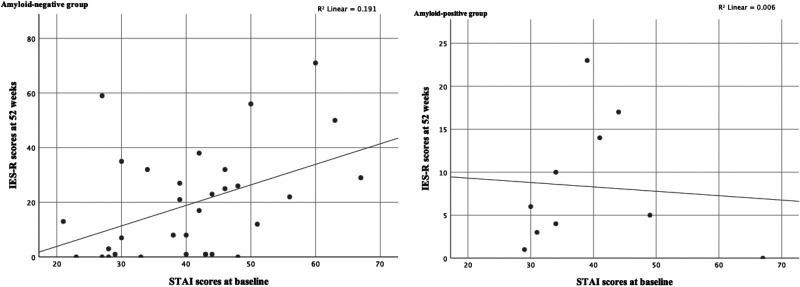
Correlations between baseline anxiety level and test-related distress at 52 weeks. IES-R indicates Impact of Events Scale–Revised; STAI, State-Trait Anxiety Inventory.

As it was suggested that individually paired change scores were relatively high at the 75th percentile in the STAI (state) and IES-R in the amyloid-negative group, further examination was conducted on correlations between baseline anxiety level and test-related distress level at 52 weeks in each group. As shown in Figure 2, Spearman’s nonparametric correlations revealed that baseline anxiety was associated with distress at 52 weeks in the amyloid-negative group (*r* = 0.40, *P* = .02), but not in the amyloid-positive group (*r* = 0.16, *P* = .66).

### Subgroup Findings in Amyloid-Negative Group

Table 4 shows that a subgroup in the amyloid-negative group (*n* = 5) had baseline STAI (state) scores that were above average compared with other participants in the amyloid-negative group (mean ± SD: 50.8 ± 7.3 vs 38.7 ± 11.2). The values in this subgroup stayed almost the same or increased over 52 weeks. Likewise, the IES-R scores in this subgroup at 52 weeks after disclosure were higher than average scores of the amyloid-negative group (mean ± SD: 43.8 ± 19.6 vs 14.8 ± 16.3).

**Table 4.** Anxiety and Test-Related Distress Among Participants in the Amyloid-Negative Group.^a^

	Subgroup With Higher Scores (*n* = 5)	Other Participants (*n* = 27)
STAI score (state)		
Baseline (predisclosure)	50.8 ± 7.3	38.7 ± 11.2
52 weeks	59.8 ± 9.7	40.8 ± 12.4
IES-R score		
52 weeks	43.8 ± 19.6	14.8 ± 16.3

Abbreviations: IES-R, Impact of Events Scale–Revised; STAI, State-Trait Anxiety Inventory.

^a^ Values are mean ± SD.

## Discussion

Disclosure of positive amyloid results to cognitively normal Japanese persons with SCD in the research setting did not appear to be associated with significant, long-term psychological harm. Scores on the STAI (state), BDI-II, and IES-R in the amyloid-positive and amyloid-negative group were within prespecified cutoff points and did not significantly change over time. Thus, receiving amyloid-positive PET results did not result in greater emotional risks compared to receiving amyloid-negative PET results. These findings are consistent with previous studies that focused on cognitively normal participants who were not distinguished from each other based on the presence or absence of SCD,^19,20^ a study on patients with mild cognitive impairment (MCI) and AD,^41^ and our preliminary study.^21^

Individual participant-level analysis (Table 2) identified 5 extremes on the high end of the distribution: 1 in the STAI (state), 2 in the BDI-II, and 2 in the IES-R. However, the scores of the remaining 2 other measurements of each participant did not change or decreased. Moreover, none of the 5 participants did not arrange follow-up visit at the memory clinic despite the aforementioned strong recommendation at enrollment by the study psychiatrists. Taken together, it was indicated that clinical significance of the extremes was doubtful.

Contrary to other studies and our expectations, however, test-related distress in the amyloid-negative group at 52 weeks after disclosure was significantly higher than that in the amyloid-positive group. Further exploration found that a high anxiety level at baseline was associated with stronger test-related distress at 52 weeks in the amyloid-negative group, whereas no association was observed in the amyloid-positive group.

Given that there were large variances in IES-R scores in the amyloid-negative group, cognitively normal older adults with SCD may potentially exist on a continuum or spectrum of awareness of cognitive decline. At one end of the spectrum, they have appropriate sensitivity regarding their cognitive decline. For example, cognitively normal older adults with SCD might be able to recognize subtle changes in their cognitive decline that were not detected by conventional neuropsychological tests. Their amyloid imaging results are likely to be positive in reflection of their “hunch.” Those who were in the middle of the spectrum have a reasonable level of anxiety. They receive amyloid-negative results with a sense of relief as measured by decreased scores on the STAI, BDI-II, and/or IES-R after disclosure. At another end of the spectrum, cognitively normal older adults with SCD may have a certain type of excessive health anxiety, or so-called disproportionate “anticipatory dementia,”^42^ “dementia worries,”^43^ or “amnesi-phobia.”^44^

The potential SCD spectrum could be supported by repeated findings that SCD reflects psychoaffective and/or personality traits.^9,45,46^ Prior research indicated that SCD was related to neuroticism,^47^ which is strongly linked to anxiety.^48^ In the current study, amyloid-negative results did not yield relief for a subgroup of 5 participants with higher STAI scores at baseline and 52 weeks and higher IES-R scores at 52 weeks. Clinicians need to be aware of those who have excessive worry about cognitive functioning out of proportion to their natural course of aging. It should be noted, however, that SCD can precede other dementia subtypes including vascular dementia, dementia with Lewy bodies, and frontotemporal dementia.^49^ In that case, an alternative potential explanation for “no relief” of participants is that they had appropriate sensitivity regarding their gradual onset of symptoms. Also, as mentioned earlier, it should be emphasized that the mean IES-R scores of both amyloid-positive and amyloid-negative participants were well below cut points for clinical significance.

We are aware that the current study presents empirical data for Japanese healthy participants for the first time regarding the long-term safety of disclosing amyloid status for predictive or diagnostic purpose. The results of this study demonstrated no significant psychological risks on cognitively normal participants with SCD with amyloid-positive PET results, and they are in line with other studies despite differences in participants’ culture or ethnicity.

The consistency among studies may partly reflect increasing interests among Japanese older adults in shared decision making, including advanced planning, that requires truth-telling.^50^ Recent reports call for immediate actions regarding the nearly 143 trillion yen ($1.3 trillion) in assets held by people with AD and other dementias in Japan.^51^ Appointing a guardian in advance to manage one’s assets is a benefit associated with learning the risks of AD before cognitive function is severely impaired. These benefits for both individuals and society may outweigh the potential harms if accumulated evidence in the future shows the value of amyloid PET as a predictive tool and conveying the information seen on these scans becomes less challenging. It is worth mentioning that performing amyloid PET on cognitively healthy adults in clinical settings is considered inappropriate in current guidelines,^52,53^ and preference sensitive decision making is required in learning the results of amyloid PET scan even in research settings.^54^

### Limitations

This study has some limitations. It is widely acknowledged that the recruitment setting (ie, memory clinics vs population based) can influence characteristics of participants with SCD.^55,56^ Higher levels of anxiety and depression in participants from a memory clinic than community-recruited healthy controls have been reported.^15^ In regard to demographic features, the age of participants with SCD varies in studies.^15,49,57^ In addition, it was reported that fear of AD was significantly greater in individuals ≥65 years than those aged 35–64 years,^58^ whereas no significant association was found in another study.^59^ Of note, the age of participants in the current study may be relatively higher than that in other studies. Thus, further studies should elaborate whether the level of anxiety of the current study represented a general population or not.

There is a lack of data for cognitively healthy older adults without SCD in the Japanese population. Thus, we were unable to evaluate whether the level of anxiety, depression, and test-related distress fell within normal limits in our study population. Furthermore, we did not assess the perceived risks for developing AD, which can contribute to the emotional impact of learning about one’s amyloid status. In light of the study design, the sample size was relatively small and the sample of amyloid-positive case was even smaller. The hospital from which participants were drawn was located in the capital of the country, which implies that the educational level of participants was slightly higher. The study protocol was unable to completely avoid self-selection bias, as participants chose whether or not to participate in the current study. Also, the mail-in approach for follow-ups after disclosure may not be able to collect sufficient information about any possible causes of the mood change in each participant. It was possible that participants could not have appropriately been excluded who had a history of schizophrenia, bipolar disorder, depression or anxiety disorder, and chronic alcohol or drug abuse/dependence at any time prior to the time period listed in the exclusion criteria. Future studies should be conducted with an attempt to minimize the potential biases.

## Conclusions

Disclosing amyloid status to cognitively healthy research participants with SCD did not cause significant long-term psychological risks. Also, there may exist a possible and theoretical spectrum of subjective concern about cognitive decline among those who receive their negative-amyloid status. There is a need to carefully identify populations that might be impacted by learning about their amyloid status by performing screening among healthy research participants enrolled in studies focusing on preclinical AD. Furthermore, future research should examine how to effectively communicate amyloid status for the risk of AD to research participants with high anxiety.
